# Influence of sequence identity and unique breakpoints on the frequency of intersubtype HIV-1 recombination

**DOI:** 10.1186/1742-4690-3-91

**Published:** 2006-12-12

**Authors:** Heather A Baird, Yong Gao, Román Galetto, Matthew Lalonde, Reshma M Anthony, Véronique Giacomoni, Measho Abreha, Jeffrey J Destefano, Matteo Negroni, Eric J Arts

**Affiliations:** 1 Division of Infectious Diseases, Department of Medicine, Case Western Reserve University, Cleveland, Ohio 44106, USA; 2 Department of Pharmacology, Case Western Reserve University, Cleveland, Ohio 44106, USA; 3 Unité des Regulation Enzymatique et Activités Cellulaires, Institut Pasteur, Paris, Cedex 15, 75724, France; 4 Department of Biochemistry, Case Western Reserve University, Cleveland, Ohio 44106, USA; 5 Department of Cell Biology and Molecular Genetics, University of Maryland, College Park, MD 20742, USA

## Abstract

**Background:**

HIV-1 recombination between different subtypes has a major impact on the global epidemic. The generation of these intersubtype recombinants follows a defined set of events starting with dual infection of a host cell, heterodiploid virus production, strand transfers during reverse transcription, and then selection. In this study, recombination frequencies were measured in the C1-C4 regions of the envelope gene in the presence (using a multiple cycle infection system) and absence (in vitro reverse transcription and single cycle infection systems) of selection for replication-competent virus. Ugandan subtypes A and D HIV-1 *env *sequences (115-A, 120-A, 89-D, 122-D, 126-D) were employed in all three assay systems. These subtypes co-circulate in East Africa and frequently recombine in this human population.

**Results:**

Increased sequence identity between viruses or RNA templates resulted in increased recombination frequencies, with the exception of the 115-A virus or RNA template. Analyses of the recombination breakpoints and mechanistic studies revealed that the presence of a recombination hotspot in the C3/V4 *env *region, unique to 115-A as donor RNA, could account for the higher recombination frequencies with the 115-A virus/template. Single-cycle infections supported proportionally less recombination than the in vitro reverse transcription assay but both systems still had significantly higher recombination frequencies than observed in the multiple-cycle virus replication system. In the multiple cycle assay, increased replicative fitness of one HIV-1 over the other in a dual infection dramatically decreased recombination frequencies.

**Conclusion:**

Sequence variation at specific sites between HIV-1 isolates can introduce unique recombination hotspots, which increase recombination frequencies and skew the general observation that decreased HIV-1 sequence identity reduces recombination rates. These findings also suggest that the majority of intra- or intersubtype A/D HIV-1 recombinants, generated with each round of infection, are not replication-competent and do not survive in the multiple-cycle system. Ability of one HIV-1 isolate to outgrow the other leads to reduced co-infections, heterozygous virus production, and recombination frequencies.

## Background

Recombination between two genetically-distinct isolates of the same retrovirus species was first described in 1970[1-3]. Retroviruses carry two copies of genomic RNA within each viral particle. Prior to a recombination event, heterodiploid viruses must be produced from cells co-infected with two different viruses. De novo infection with a heterodiploid retrovirus can then result in generation of recombinant or chimeric genomes catalyzed by reverse transcriptase jumping between genomic RNA templates[4,5]. Several groups have studied various aspects of these recombination events and have defined various possible models for retrovirus recombination involving synthesis of both the minus and plus strands of retroviral DNA[6-10]. However, increasing evidence suggest that the majority of recombination events occur during synthesis of the minus DNA strand, following a copy choice mechanism[11]. This transfer involves a jumping of the nascent DNA strand from one RNA template to the other, which is guided through local sequence similarity between the two genomic RNAs. Various triggers may be responsible for this template switching such as breaks on the genomic RNA, pause sites for reverse transcription, or particular RNA secondary structures in the viral genome[12].

**Figure 1:**
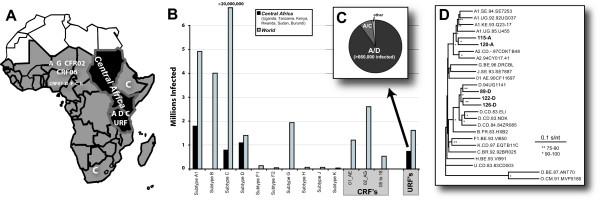
**Prevalence of unique HIV-1 recombinant forms (or intersubtype HIV-1 recombinants)**. The location of subtypes (e.g. A, C, G, etc), circulating recombinant forms (CRFs), and unique recombinant forms (URFs) are mapped in sub-Saharan Africa and specifically, ***Central Africa ***in panel **A**. The number of humans infected with the dominant subtypes, CRFs, and URFs in the world or in Central Africa is graphed in panel **B**. The proportion of specific intersubtype recombinants (A/D, A/C, and others) responsible for URF infections in Central Africa has been reported (**C**) [20–23]. Panel D provides a neighbor-joining phylogenetic tree to describe the genetic relationship of the C1 to C4 env sequences of 115-A, 120-A, 89-D, 122-D, and 126-D to other reference HIV-1 sequences.

Identification of viral encoded oncogenes provided circumstantial evidence of retroviral recombination but actual in vivo corroboration of this recombination process is most obvious in infections by human immunodeficiency virus type-1 (HIV-1). HIV-1 recombination appears rampant during infection and may be a major evolutionary mechanism responsible for shuffling of genetic markers[13,14]. Unlike this intrapatient recombination, shuffling of divergent HIV-1 regions and the creation of chimeric genomes is now apparent throughout this epidemic[15-17]. HIV-1 has evolved and diversified in the human epidemic into three groups (M, N, and O) and at least ten subtypes (A through J) within the predominant group M[18,19]. In East Africa and specifically Uganda, subtypes A and D of HIV-1 group M co-circulate with a high prevalence (50% subtype A, 40% subtype D) [20,21]. Co-circulation of subtypes gives rise to unique recombinant forms (URF) such as A/D recombinants in Uganda but continual human-to-human transfer of recombinants with defined mosaic genomes has lead to the identification of circulating recombinant forms (CRF01 to CRF16) [15]. Interestingly, A/D URF as opposed to stable CRFs are predominant in Uganda [20,21]. The impact of URFs is obviously increasing with the merger and expansion of regional epidemics with divergent subtypes (Figure 1A and 1B). Underestimates suggest that nearly one million individuals are infected with URFs (Figure 1B) and that intersubtype A/D recombinants in Central Africa are estimated in 660,000 of this million (Figure 1C) [20-23].

The frequency of recombination within the 9.7 kilonucleotides of HIV-1 genome fluctuates between three and thirty recombination events per round of replication and depending on use of various viral genomes and possibly, the cell type for infection[9,24,25]. In general, these recombination frequencies are typically derived from experiments employing closely related or even identical parental sequence. Few studies have employed non-subtype B sequences or actual pairs of HIV-1 isolates that recombine and circulate in the epidemic. Two studies have examined intersubtype recombination in the 5' untranslated region[26,27]. Increased sequence homology and maintenance of dimerization initiation sequence appeared to stimulate intersubtype recombination employing an in vitro reconstituted reverse transcription system[27] and a single cycle replication system[26].

In this study, we generated recombinants between subtypes A and D in the C1-C4 region of the envelope gene using three different assay systems. Two subtype A and three subtype D primary HIV-1 isolates from Uganda were employed as the "base" virus or env sequence for these recombination frequency analyses[28]. The in vitro reconstituted system (referred to as in vitro system) involves RNA-dependent DNA synthesis catalyzed by HIV-1 reverse transcriptase employing purified RNA templates of subtype A and D *env *C1-C4 regions[29]. The second assay employs a single cycle tissue culture infection with defective HIV-1 particles (referred to as single cycle system)[30], while the third system requires multiple round infection of susceptible cells with two HIV-1 isolates of subtypes A and/or D (referred to as multiple cycle system)[31]. Intra and intersubtype recombination frequencies in the *env *gene were calculated from all three systems. For the in vitro and single cycle systems, recombination frequency was calculated from the conversion of lac- (parental) to lac+ (recombined) phenotype[29,30]. In the multiple cycle system, replication-competent parental versus recombined HIV-1 isolates (i.e. in the *env *gene) were selectively PCR amplified with subtype-specific or isolate-specific primers in order to calculate recombination frequency[31]. In general, increases in genetic diversity between the HIV-1 *env *gene templates results in decreased recombination frequencies but there are exceptions to this observation. It appears that strong hotspots for recombination can appear with select pairs of HIV-1 isolates and may be dependent of specific nucleotide sequence/structure combinations between donor/acceptor templates. A previous study mapping these recombination breakpoints[32] assisted in our analyses of their impact on intersubytpe recombination frequencies. In vitro analyses were performed to examine the impact of unique recombination hotspot in the C3/V4 region of *env*.

## Results

### Intra- and intersubtype recombination frequency after a single cycle of infection

**Figure 2:**
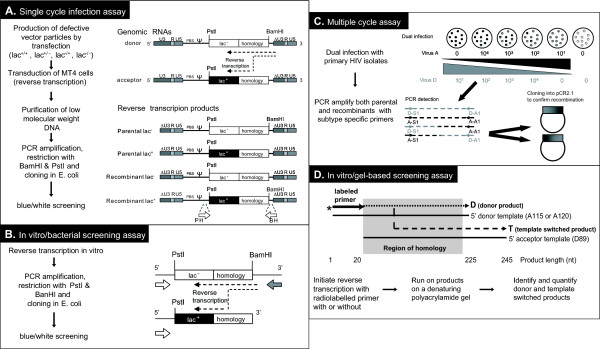
**Schematic representation of intra- and intersubtype recombination systems**. Single cycle tissue culture system (panel **A**) for recombination employed heterozygous VSV-pseudotyped *env *particles produced by transient co-transfection of two genomic and two helper plasmids in 293T cells. Following production from 293T cells, virus particles were used to transduce MT4 cells. PCR products cleaved with BamHI and SacII were then cloned into vectors for transfection into E. coli followed by screening of blue and white colonies. Calculations for the frequency of recombination are outlined in the Materials and Methods. Structure of the genomic plasmids and reverse transcription products are shown in panel **A**. The in vitro experimental system is outline in panel **B **and involves reverse transcription across a donor RNA template that shares a region of homology with an acceptor RNA template upstream of a genetic marker (*lacZ'*) on the acceptor RNA or a truncated, non-functional portion of the *malT *gene from *E.coli *on the donor template. The donor RNA also contains at its 3' end an extension which is used to selectively prime reverse transcription after hybridization of a complementary oligonucleotide. Processive copying of the donor template will yield *lac*^- ^genotypes, while template switching during reverse transcription of the retroviral sequence will produce *lac*^+ ^genotypes. The resulting double-stranded DNAs are restricted with *Bam*HI and *Pst*I and, after ligation to a plasmid vector, used for bacterial transformation. On appropriate media, recombinant DNAs will yield blue colonies distinguishable from the white colonies given by the parental DNAs. The same LacZ screening system is employed for single cycle assay (**A**). Multiple cycle tissue culture system (panel **C**) was performed by infecting U87.CD4.CXCR4 cells with subtype A and D HIV-1 isolates in pairs (0.001 MOI). After the first round of replication, co-infected cells can produce both parental and heterodiploid viruses. Infection of new cells with heterodiploid virions can lead to intersubtype recombination. The schema for PCR amplification of intersubtype HIV-1 *env *fragments is outlined in panel **C **and the calculation for frequency is described in the Materials and Methods. Finally, panel **D **describes the reconstituted in vitro reverse transcription assay which involves initiating HIV-1 DNA synthesis from a radiolabeled DNA primer annealed to a defined donor RNA template (e.g. C3-V4) and in the same reaction mixtures with an acceptor RNA template slightly longer and with a region of sequence homology with the donor to promote strand transfer. RNA-dependent DNA synthesis is catalyzed by reverse transcriptase and a 20 nt 5' [^32^P]-labeled DNA primer on the RNA donor templates (225 nt), RNA acceptor templates (225 nt), and with or without NC. The templates have a 205 nt overlap region to promote intersubtype recombination. Products from these reactions were resolved on a 8% denaturing polyacrylamide gel.

As schematically illustrated in Figure 2A, we constructed HIV-1 genomes which contained the *env *gene of HIV-1 subtypes A and D isolates (115-A, 120-A, 89-D, 122-D, and 126-D) downstream of Lac Z- (donor genome) or Lac Z+ (acceptor genome). Defective retroviral particles were produced by co-transfections of the genomes into a 293T cell packaging line. The *env*/lac Z cassette was than PCR amplified following single-cycle infection with heterozygous and homozygous VSV-pseudotyped HIV-1 particles. As described in Figure 2, the reverse transcription products resulting from processive copying of the donor RNA and those generated by template switching in the region of homology (the PstI-BamHI products; Figure 2A) were cloned into plasmids for blue (lac+)/white (lac-) screening of E. coli colonies (see Materials and Methods). This experimental system has been previously described as a method to study HIV-1 copy choice recombination after a single cycle of infection of human cells [30]. For each experiment, a control sample was run in which homozygous lac^+/+ ^and lac^-/- ^defective viruses were produced separately by transfection of 293T packaging cells with either pLac^- ^or pLac^+ ^genomic plasmids. The frequency of blue colonies, following cloning of the PstI-BamHI products, provides an estimate of the background (non-RT generated) recombinant molecules (see Materials and Methods). These recombinants could have been generated by the Taq polymerase jumping between the templates. However, the frequency of these background recombinants was always lower than 0.5%, or at least 20-fold less than the HIV-1 recombination frequency obtained with heterozygous virions (data not shown)).

**Figure 3:**
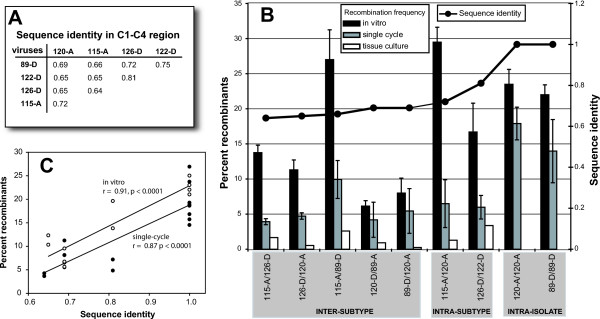
**Frequency of inter- and intrasubtype HIV-1 recombination in an in vitro, single cycle, and multiple cycle assay systems**. Panel **A **indicates the nucleotide genetic distances that separate the *env *genes in each pair of subtype A and D primary HIV-1 isolates employed in this study. The recombination frequencies of each pair in panel **B **were calculated in three systems. For in vitro, the synthesis of minus strand DNA on the donor RNA template was catalyzed by RT. Products were PCR amplified, cloned, and blue/white colonies were screened to calculate recombination frequencies. For the single-cycle system, recombination occurred in a cell infected with a heterozygous virus particle. Recombinants were identified by PCR and by the same blue/white colony screening described in Figure 1 and in the Materials and Methods. Finally, the recombination frequency in the multiple cycle system was measured by quantitative PCR using isolate- or subtype-specific primers (see Materials and Methods and Figure 4). The sequence identity between each HIV-1 pair is shown as line graph with the scale on right of panel **B**. Panel **C **shows a plot of recombination frequency in the single cycle (filled circle) or in vitro (open circle) systems versus sequence identity. The r value represents the Pearson product moment correlation for in vitro (open circle) and single cycle (filled circle) assays.

The sequence identity in this *env *fragment ranged from 0.676 to 0.734 between the subtype A and D isolates and 0.794 to 0.815 within isolates of the same subtype (A or D). A neighbor-joining phylogenetic tree describes the genetic relationship between these subtype A and D HIV-1 isolates and other reference strains (Figure 1D). The pairwise distances between each isolates is shown in Figure 3A. Using the single cycle tissue culture assay, there was an increased frequency of recombination corresponding with increased sequence homology (Figure 3B and 3C). The intersubtype A/D and D/A pairs recombined with a frequency between 3.9 to 5.5% (with the exception of 115/89) while the intrasubtype pairs recombined with a frequency of 5.9 and 6.5%. This single-cycle system employs a lacZ reporter gene for the detection of recombination events occurring upstream in the sense of (-) strand DNA synthesis[32]. Thus, it is possible to measure recombination frequency due to jumping between identical template sequences from the lacZ- to the lacZ+ template (see Figure 2A). The intra-isolate frequency of recombination ranged from 13.9 to 17.7% in this assay (Figure 3B).

**Figure 4:**
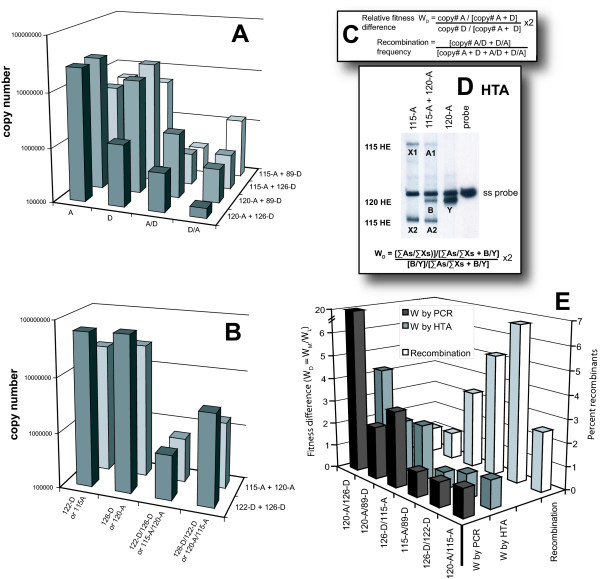
**Measuring fitness and recombination frequency in the multiple cycle system**. U87.CD4.CXCR4 cell cultures dually infected with two isolates of different subtypes (A+D; panel **A**) or the same subtype infections (A+A or D+D; panel **B**) and then harvested for analyses. Subtype or isolate-specific primers were employed to amplify parental or recombinant HIV-1 *env *DNA (X axes) from specific dual infections (Z axes). Copy numbers on the Y axes were derived from control PCR amplifications with known copy numbers of subtype A and D DNA templates (10^2 ^to 10^8 ^copies/reaction) (see Materials and Methods). Relative fitness values (W) and frequencies of recombination from these dual infections/competitions were calculated as described in the inset of panel **C**. Briefly, conserved primers were utilized to PCR amplify the *env *genes from parental and recombinant *env *progeny from each dual infection to measure fitness by HTA[54,62,63]. These PCR products were then denatured and annealed to a radiolabeled *env *probe, which was amplified from a subtype E HIV-1 *env *clone (E-pTH22. DNA heteroduplexes specific for the each parental isolate were resolved on a 6% nondenaturating polyacrylamide gel. A sample autoradiograph and calculations of relative fitness is defined in panel **D**. A plot of the fitness differences (W_D _= W_more fit_/W_less fit_) or of percent recombinants (right Y axis) for each dual infection pair is shown in panel **E**.

When the 115/89 pair was excluded from the analyses, there was significant correlation between the recombination frequency and sequence identity between donor and acceptor templates (r = 0.87, p < 0.0001; Pearson product moment correlation) (Figure 3C). As described below, we identified a strong hotspot for recombination in the C3 *env *regionwhen the 115-A was employed as donor template in this single-cycle system (ref). This hotspot for recombination was not observed with any other donor/acceptor template pair and appeared unique to 115-A template as donor. The impact of this 115-A specific hotspot on high recombination frequencies is explored below.

### Recombination frequency in vitro

In order to compare the results of the single cycle cell culture assay to an in vitro assay, the same 1100 nucleotide region of the HIV-1 subtype A and D *env *genes were cloned into pA and pK vectors as previously described. In the cell-free assay outlined in Figure 2B, reverse transcription is primed on the donor RNA in the presence of the acceptor RNA. Presence of a functional lacZ gene indicates a strand transfer from donor to acceptor RNA. As with the assay in cell cultures, the position of template switching in the region of homology is detected by sequence analysis after cloning the products of reverse transcription. Using this in vitro reverse transcription assay, the frequency of recombination with the intersubtype pairs generally ranged from 6–13.8%, with the exception of the A-115/D-89 pair, which had a recombination frequency of 27%. Use of intrasubtype pairs as with the single-cycle system resulted in higher recombination frequencies: 16.7% with D-126/D-122. Again the 115-A/120-A pair was the exception to this observation with a recombination frequency of nearly 30%. Expectedly, the highest recombination frequencies were observed when the same isolate was used in the donor and acceptor RNA templates. The intra-isolate pair D-89/D-89 recombined with a frequency of 22%, and the 120-A/120-A pair recombined with a frequency of 23.5%. As with the single cycle assay there was a significant but proportional increase in recombination frequencies with increasing sequence identity between donor and acceptor templates (Figure 3C). In all cases, the frequency of recombination was higher in the in vitro reconstituted reverse transcription assay than in the single-cycle assay. Increased frequency of recombination in vitro can be misleading considering the inability to reconstitute the conditions of endogenous HIV-1 reverse transcription. Compositions of buffers, concentrations of substrates (e.g. templates and dNTPs), and the amount of reverse transcriptase is optimized for DNA transcription and does not necessarily reflect the actual native components or concentration levels. Equal ratios of acceptor and donor templates were however employed in vitro to mimic in vivo conditions.

### Frequency of intersubtype recombination in a multiple cycle assay

A PCR method relying on subtype-specific oligonucleotides was devised [31] to detect, amplify, and quantify *env *recombinants in HIV-1 dual infections. In the case of intrasubtype dual infections, it was necessary to design new isolate-specific primers for amplification of recombinant *env *genes. Amplification of recombinant *env *genes with subtype or isolate-specific primers was preceded by an external PCR amplification using conserved *env *primers (Figure 2C). In this experiment, plasmids containing entire *env *gene of the parental or recombined A/D viruses (pA-*env*, pD-*env*, pA/D-*env*, and pD/A-*env*) were employed as PCR amplification controls. Briefly, the control plasmids were serially diluted, PCR amplified, and used as standard curves to determine copy number of PCR-amplifed recombined viral RNA molecules derived from the dual infections. This method of quantitative PCR amplification has been previously reported[33,34]. As expected, the subtype A-specific (or isolate-specific) primers amplified *env *DNA from pA-*env *control plasmid, mono-infection and dual infections containing A virus but failed to amplify product from the D mono-infection. Similar findings were obtained with the subtype D-specific primer pair.

To measure the frequency of intra- and intersubtype recombination during multiple rounds of replication, a 10^-2 ^or 10^-3 ^multiplicity of infection (MOI) of the A or D isolates were added in pairs to U87.CD4.CXCR4 cells. Dual infections were monitored each day and when peak reverse transcriptase (RT) activity was detected in the supernatant, virus was harvested and subject to RT-PCR. HIV-1 recombinants and parentals were then PCR amplified with subtype (isolate)-specific primer sets as described above. After correcting for difference in primer annealing and amplification efficiency using the plasmid controls, the copy number of the parental isolates and *env *recombinants amplified from these dual infections were plotted in Figure 4A (intersubtype competitions/recombinations) and Figure 4B (intrasubtype competitions/recombinations). Based on division of recombined C1-C4 products by the total C1-C4 products (parental plus recombined), we estimated that the frequency of inter- and intra- subtype recombination in the *env *fragments ranged from 0.25 to 3.4% (Figure 4E). To control for recombination generated by Taq, equal amounts of both pA-*env *and pD-*env *plasmids (10^3 ^or 10^6 ^copies/reaction) were added to PCR amplifications employing the subtype or isolate-specific *a*-envC1/*a*-envC4, *d*-envC1/*d*-envC4,*d*-envC1/*a*-envC4, and *a*-envC1/*d*-envC4 primer pairs. Only the *a/a *and *d/d *primer pairs could efficiently PCR-amplify the mixture of the pA and pD plasmids [31]. The frequency of recombination catalyzed by Taq was < 0.005%/Kbp, or at least 100-fold less than that generated in dual infections of U87.CD4.CXCR4 cells.

In the multiple cycle assays as with the in vitro and single cycle assays, the recombination frequencies appeared to be proportionally higher with the intrasubtype pairs than with the intersubtype A/D pairs (Figure 4E). However, there was no significant correlation between recombination frequency in the multiple cycle system and sequence identity between virus pairs (data not shown). A marked decrease was observed in the overall recombination rates in the multiple cycle tissue culture assays (range from 0.25 to 3.4%) as compared with the single cycle (4–17%, p < 0.005) or in vitro assays (6–30%, p < 0.001). This decrease in recombination frequencies over multiple rounds of replication appears counterintuitive considering each round of replication of both parental viruses would increase chances of a co-infected cell and of heterodiploid virus production. These heterodiploid viruses are the progenitors of intersubtype (or intra-) recombinants upon de novo infection. Furthermore, the recombined viruses can also produce progeny to infect new cells and expand in culture. This is of course assuming that all recombinants are not defective and are of equal fitness as the parental isolates. Unlike the single cycle assay, the recombined *env *glycoproteins at the time of virus production will only show functional constraints when infecting a new cell. Thus, it seems unreasonable to assume that all recombined *env *genes, generated by reverse transcription, will be functional or will mediate host cell entry with equal efficiencies[31].

### Comparing the relative virus production in a dual infection with recombination frequency

Relative production of both viruses in a dual infection can be measured and compared to the frequency of recombination. The *env *gene is PCR amplified with conserved *env *primers from the dual infection and then submitted to heteroduplex tracking assay (HTA). Quantitation of the segregated heteroduplexes on the non-denaturing polyacrylamide gels estimates production of each virus from the dual infection. Figure 4C provides an example of the HTA analyses and relative fitness calculation. The fitness difference (W_D_; left y-axis of Figure 4E) between the two viruses is a ratio of the relative fitness values of the more fit over the less fit virus produced from the dual infection/competition. A fitness difference of 1 suggests equal replicative fitness between the pair of viruses. Relative fitness values in these competitions can also be calculated using the production of each virus (Figure 4C) as measured by quantitative PCR (Figure 4A and 4B). As indicated in Figure 4E, the fitness difference between two HIV-1 isolates in competition as determined by quantitative PCR was nearly identical to those values calculated by HTA. Relative fitness values for this study were derived from dual virus competitions in the U87.CD4.CCR5 cultures. Nearly identical relative fitness values were obtained from competitions in PHA/IL-2 treated PBMCs [28]. For example, the fitness difference between 120-A over 126-D in U87.CD4.CXCR4 cultures was 4.25 and 3.04 in PHA/IL-2 treated PBMC cultures (as determined by HTA) [28].

When comparing relative fitness (left y-axis, Figure 4E) and recombination frequency (right y-axis), it is quite apparent that the ability of one virus to out compete the other in a dual infection dramatically reduces the frequency of recombination. In contrast, equal fitness of both viruses in cultures results in the highest recombination frequency. For examples, a four-fold increase in virus 120-A over virus 126-D production in a dual infection was associated with recombination frequency of less than 1%/Knt whereas equal fitness of 126-D and 122-D (W_D _= 1) in a dual infection resulted in a higher frequency of intersubtype recombination (6.5%/Knt). This finding was consistent in all dual infections. Equal replication efficiency in a dual infection (i.e. equal fitness) would result in a higher likelihood that both virus types can co-infect more cells, leading to a greater production of heterozygous virions (containing two different genomes), and thus, a higher frequencies of recombination.

It is important to note that following infection with heterozygous virions, the rate of recombination events in the multiple cycle/dual infection is likely similar to that observed in the single-cycle assay. Over multiple rounds of replication and in the absence of selection, there should be an increase in the amount of dually infected cells, production of heterozygous virions, and as a consequence, the production of recombined viruses. Thus, the apparent reduction in the frequency of recombination (compare multiple to single cycle frequencies, Figure 3B) is likely related to the high proportion of replication defective or dead HIV-1 recombinants following each round of reverse transcription/template switching.

### Increased recombination rates with 115-A donor template

**Figure 5:**
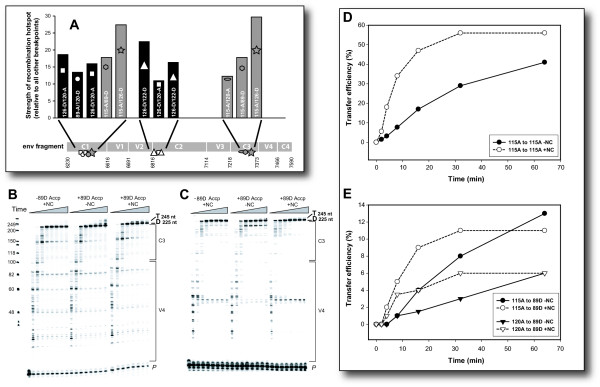
**Pausing patterns and hotspots of intersubtype recombination during reverse transcription on the 115-A and 120-A RNA donor template**. Hotspots of recombination were mapped in the C1-C4 region as part of a previous study (**A**). RNA-dependent DNA synthesis is catalyzed by reverse transcriptase and a 20 nt 5' [^32^P]-labeled DNA primer on the 115-A and 120-A RNA donor templates (225 nt). Reactions were in the presence or absence of D-89 acceptor (225 nt) and NC (Figure 1D). Reactions were stopped at 30 s, 1, 2, 4, 8, 16, 32, and 64 min and run on a 8% denaturing polyacrylamide gel. Products of these reactions are shown in the autoradiographs of panel **B **(A-115 donor) and panel **C **(A-120 donor). The positions of the primer (P), and full extended products derived from the donor template (D, 225 nt) and the strand transfer products (T, 245 nt). A major pause site during DNA synthesis was observed in the C3 region of 115-A donor template and is indicated by the "dumbbell" symbol. A putative V4 stem-loop is also outlined (see Figure 5 for details). Graphs of transfer efficiency vs. time for reactions with A-115 as both donor and acceptor (panel **D**) or A-115 (circles) or A-120 (triangles) as donor and D-89 as acceptor (panel **E**) are shown. Filled shapes are without NC and open with. The % transfer efficiency is defined as the amount of transfer product (T) divided by the sum or transfer plus full-length donor directed (D) products times 100 ((T/(T + D)) × 100).

As described above, the recombination frequencies with 115-A as donor were significantly higher than with other subtype A and D RNA donor templates in both the in vitro and single-cycle systems. Furthermore, 115-A as donor was exception to the direct relationship between increasing recombination frequencies with increasing sequence identities between acceptor and donor templates. Explanations for this exception were explored by investigating comparing the sites of recombination in the C1-C4 *env *region. A thorough analysis of recombination breakpoints in Baird et al[32] revealed that most intrasubtype and intersubtype *env *recombinants had preferential cross-over sites in the C1 region and V2/C2 junction of *env *(Figure 5A). However, the use of the donor 115-A template with any other acceptor template resulted in a unique recombination hotspot at the junction of the C3/V4 region (Figure 5A). Considering the C3 breakpoint was responsible for one third of all 115-A-derived *env *recombinants, it is possible that this additional hotspot led to a significant increase in recombination frequency. Aside from this 115-A-specific *env *C3/V4 recombination site, the distribution of recombination sites with all the HIV-1 template/virus pairs were quite similar across the C1 to C4 region.

To further investigate this C3 recombination site, we analyzed the pausing pattern and template switching frequency in the C3 to V4 regions employing a reconstituted in vitro reverse transcription assay described in Figure 2D. Minus strand DNA synthesis was catalyzed by HIV-1 RT and primed from a radiolabeled primer annealing to the 115-A or 120-A donor RNA template (Figure 5A and 5B, respectively). Template switching to the 89-D template during minus strand DNA synthesis was monitored during a time course assay and in the presence or absence of HIV-1 nucleocapsid protein (NC) (schematic, Figure 2D). The addition of 5' non-homologous nt's to the 5' end of the donor prevents transfer from the end of the donor and thus, limits transfer to the boxed region in the schematic diagram (Figure 2D). Approximately 50% of the minus strand DNAs were chased to full-length product derived from the donor template (225 nt D product; Figure 5B and 5C). There does appear to be more RT pausing on the 115-A template as compared to the 120-A template during (-) strand DNA synthesis and specifically in the C3 region of the template (indicated in Figure 5B). This pause product was observed when donor template was present or absent in the reaction mixture indicating it originated from the (-) strand DNA synthesis off the 115-A donor tempate. Most of the paused products were eventually elongated during the time course reaction.

A small percentage of minus strand DNA jumped to the acceptor template for continued elongation (245 nt T product; Figure 5B and 5C). Using primers specific for the strand transferred minus strand DNA products, we PCR amplified the recombinants and sequenced 35 clones from the 115-A/89-D assays. Recombination in this reconstituted in vitro assay generally matched those C3-V4 recombination sites observed in the single-cycle assay involving the same pair (data not shown). Interestingly, the presence of NC in the reactions led to an even more pronounced focus of breakpoints in C3 region. NC is known to increase the frequency of recombination, possibly through the destabilization of RNA structures to increase strand invasion and transfer. The frequencies of strand transfer events along these templates were plotted in Figure 5D and 5E. As observed in the in vitro and single cycle assays (Figure 3B), increased sequence identity between donor and acceptor RNA templates appears to augment recombination or strand transfer during reverse transcription. When 115-A RNA was employed as both donor and acceptor, the strand transfer efficiency reached levels of nearly 40% without NC and 60% with NC (Figure 5D). In contrast, transfer efficiency with the donor 115-A/acceptor 89-D intersubtype pair was less than 14% even with NC (Figure 5E). The sequence identities for the 115-A/89-D pair and the 120-A/89-D pair were nearly identical at 0.66% and 0.69, respectively (Figure 3A). However, the transfer efficiency from the 115-A to the 89-D templates was at least 2-fold greater than the transfer efficiency from the 120-A to the 89-D templates. The addition of NC to the reactions proportionally increased transfer efficiency with both template pairs. In other words, increased transfer from 115-A to 89-D than from 120-A to 89-D was apparent throughout the time course with or without NC. These results again suggest that the preferential C3 breakpoint in the 115-A donor template (absent in 120-A donor template) is increasing recombination frequency.

**Figure 6:**
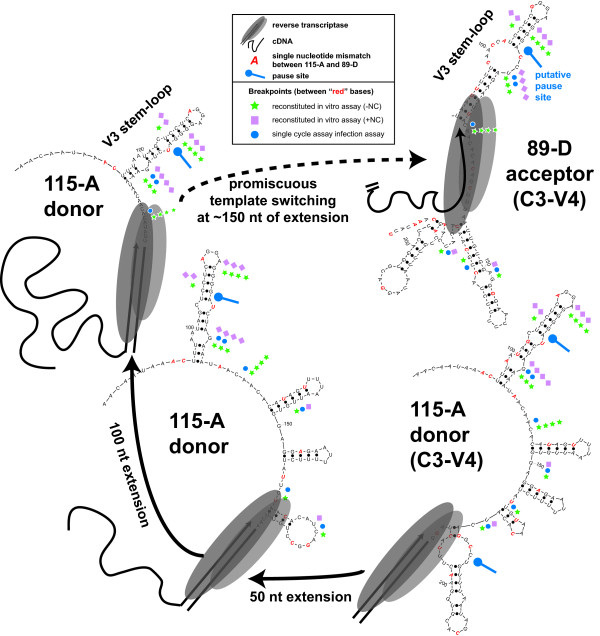
**Schematic of reverse transcription and template switching from the 115A to the 89D RNA templates**. A 350 nt RNA sequence from position 7150 to 7500 (HXB2 numbering) of virus 115A, 120A, 89D, 122D, and 126D was submitted to Mfold server at MacFarlane-Burnett for RNA structure prediction based on the Zucher algorithms. The RNA structures for the five different templates varied considerably but one stem-loop or hairpin was consistently present around nt 7301 to 7339 and was termed the V4 stem-loop. Only 115A and 89D RNA structures between nt 7296 and 7421 are presented in this figure. Shifting the 350 nt RNA template 50 nt upstream or downstream did not affect the structure of this predicted V4 stem-loop. This RNA structure also contains the strong pause site identified in Figure 4B (blue dumbbell). The structure of virus 115A RNA was examined by sliding a 50 nt window from nt 7276 to 7126 on the 3' end and from nt 7235 to 7075 on the 5'end. To mimic the double stranded DNA/RNA duplex generated by (-) strand DNA synthesis by reverse transcriptase, a 20 nt duplex was added into the submitted sequence at the 3' end (designated as twenty "X's" in the sequence). The progression of RT on the 115-A RNA template for a 50, 100, and 150 nt extension is illustrated. The promiscuous template switching event to the 89-D template, near V4 stem-loop is also depicted. The "red" nucleotides represent the mismatched sequences between 115-A and 89-D templates. Recombination sites were identified and mapped to the sequences between these base mismatches. The "green stars" represent the recombination breakpoints identified in 16 recombinant clones from the reconstituted in vitro assay in the absence of NC (Figure 4B) and "pink squares", those in 18 recombinant clones from the reconstituted in vitro assay in the presence of NC (Figure 4B). The "blue circles" represent the breakpoints from five clones of the single cycle infection assay, i.e. identified in the C3-V4 region).

The mechanism(s) for the increased C3/V4 recombination frequency when 115-A RNA was acting as the donor is under investigation. Preliminary data suggest that specific sequence and RNA folding of the 115-A as compared to other subtype A and D templates may play an important role in directing strand transfer during reverse transcription in the C3/V4 region (Figure 6). To investigate if RNA sequence and secondary structure may play a role in the C3/V4 breakpoint selection, a 350 nt RNA sequence encompassing the C3-V4-C4 region was folded using Mfold and the new Zucher algorithm. Computer predictions indicate that the RNA structures were not conserved between the subtype A or D RNA templates (data not shown). One stable RNA stem-loop (termed V4 stem-loop) was, however, found between nt 7301 and 7339 on the 115-A template (Figure 6) as well as the other viral sequences, even though this region is fairly heterogenous. This structure was maintained even when RNA structures were predicted with a 50 nt sliding window (Figure 6) and when folding larger env RNA sequences (data not shown). The 50 nt sliding window removed 3' RNA sequence on 115-A to simulate the RT moving along the RNA template during RNA-dependent DNA polymerization. The "movement" in the 5' direction on the template could result in the re-folding of RNA and the destabilization of some RNA structures. However the V4 stem-loop remained intact. Sequence analyses of the 115-A/89-D recombinants from in vitro reverse transcription assays (from Figure 5) and from the single cycle infection assays [27] indicate that breakpoints were clustered in this V4 stem-loop (Figure 6). Breakpoints could be mapped to specific regions flanked by mismatched bases between the 115-A and 89-D RNA templates (Figure 6). A reverse transcription pause site was also mapped to the middle of the 3'end of this stem-loop (Figures 5B and 6). As outlined in the discussion, a similar stem-loop configuration and its contribution on preferential recombination were described for the C2 region of HIV-1 [24].

## Discussion

This study has explored the mechanisms of intra- and intersubtype HIV-1 recombination using primary subtype A and D isolates that co-circulate and recombine in Uganda. Retroviral recombination originates from two different virus isolates co-infecting a single cell and then producing heterodiploid retrovirus particles. Upon de novo cell infection, reverse transcriptase jumps between the two heterologous genomes during (-) strand DNA synthesis and creates a chimeric proviral genome. A significant proportion of this recombined progeny may be dead, defective, or less fit than the parental retrovirus isolates. Those recombinants that do survive and compete may have a selective advantage. In terms of the HIV-1 epidemic, the survival and transmission of intersubtype HIV-1 recombinants can represent major antigenic shift and possibly changes in virulence [16,35]. Within an infected host, recombination is a rapid form of evolution that likely contributes to immune evasion and multi-drug resistance [13,14].

The mechanisms controlling the generation of intersubtype HIV recombinants are poorly understood but the subject of intense investigation [24-26]. The majority of earlier studies have focused on retroviral recombination in regions presenting a high sequence similarity and not necessarily with HIV or even retroviral sequences [5,10,36-40]. In this study, we have employed either primary subtype A and D HIV-1 isolates or their *env *genes cloned into retroviral/RNA expression vectors. We then compared the frequency of recombination during reverse transcription in vitro, a single cycle infection, and following multiple rounds of virus replication. Both the in vitro and single-cycle system measure RT specificity for strand transfer/recombination in the *env *gene but do not measure the production or selection of functional envelope glycoproteins. The multiple cycle system involves infecting susceptible cells with two HIV-1 isolates of the same or different subtypes. The frequency of recombination in this system is a function of cell co-infection frequency, heterodiploid virus production, recombination rates within an infected cell, and finally selection of replication-competent and fit intra- or intersubtype recombinants.

Findings from all three assay systems suggest that recombination frequency is significantly reduced with decreasing sequence identity between the virus (or RNA template) pairs, confirming previous observations on intersubtype recombination [39] and on recombination between HIV-1 templates with engineered sequence diversity[40,41]. The relationship between recombination frequency and sequence similarity is not an absolute as indicated by intersubtype recombinations involving the subtype A 115 RNA as donor. Concurrent studies have carefully mapped the recombination breakpoints derived from all of these intra- and intersubtype pairs and in all three systems[32]. Increased recombination frequencies when 115A donor RNA was paired with any other acceptor RNA template (subtype A or D) appears related to a unique V4 *env *"hotspot" for recombination. A reconstituted in vitro reverse transcription system was employed to dissect the mechanism(s) of enhanced strand transfer from the 115-A V4 region. Preliminary data suggests that specific RNA secondary structures in the V4 RNA may have driven this preferential strand transfer and increased recombination frequency. This type of observation may have escaped the attention of earlier reports [9,24,40,42] since the identification of these heterogenous recombination hotspots requires considerable RNA sequence diversity.

The C3/V4 hotspot for recombination may be driven by pausing of reverse transcriptase on the 115A template, which may be absent with the other RNA donor templates. The mechanism for preferential recombination at this V4 stem-loop may be related to that observed at a similar recombination hotspot in the C2 region of *env *[24]. We are now exploring if a specific signature sequence and RNA structural element was required for the 115-A-mediated strand transfer, as opposed to more random strand transfer from the other HIV-1 RNA templates in this region. A stem-loop structure may be present and promote recombination in the conserved regions of the *env *genes of multiple HIV-1 subtypes. However, only the appropriate sequence combination can generate similar hairpins in a hypervariable region of the donor and acceptor RNA templates, and as consequence promote preferential recombination. This combination of sequence/structure could also skew the direct relationship between relative sequence identity and recombination frequency.

This study provides the first evidence that HIV-1 subtype A and D isolates, found to co-circulate in Uganda [20,21,43], can recombine in tissue culture (ex vivo) but less efficiently than two isolates of the same subtype. As described, selection for replication competent HIV-1 recombinants will likely result in an apparent decrease in recombination frequencies generated in cells infected with a heterozygous virion. In addition, differences in the replicative fitness of the two primary HIV-1 isolates used for co-infection will affect the rate of recombination [35]. Fitness is a complex parameter defined by replication capacity in a given environment [44]. In terms of dual infection of susceptible cells, increased replicative fitness of one HIV-1 isolate over the other reduced the frequency of co-infected cells, heterodiploid virus production, and as a consequence, recombination frequency. It was quite apparent from our results that increased replicative fitness of one HIV-1 isolate over the other in the dual infection reduced recombination frequency. The relevance of this observation can be quite profound on recombination within a dually infected individual considering even modest differences in replicative fitness can dramatically decrease recombination frequency. Within this dually infected individual, fitness differences rather than the genetic differences between the infecting isolates can have a greater impact on recombination.

Regardless of the impact of fitness, recombination frequencies in the multiple cycle system were still 5- to 10-fold less than that observed in the single cycle system. This observation is counter-intuitive due to the expected increase in circulating recombinant forms with ongoing co-infection of cells and recombination. Therefore, it quite reasonable to assume that recombination between diverse HIV-1 *env *genes can result in non-functional envelope glycoprotein and as a consequence, replication defective or non-viable virus. We are currently examining the proportion of viable, defective, and dead envelopes in the three systems by cloning the products into expression vectors and assessing their function in cell fusion assays. In addition, we are developing a mathematical model that assumes all HIV-1 recombinants are viable in a multiple cycle/dual infection assay. This model accounts for differences in replicative fitness, probability of mono- and dual infections (represented by Poisson distributions), the relative production of homozygous versus heterozygous virions, frequency of recombination (derived from the single cycle assays), and virus progeny production (both parental isolates and recombined viruses). Preliminary data suggests that over 75% of the HIV-1 recombinants in a dual infection are not replication competent and do not contribute to the recombination frequency in the multiple cycle system.

## Conclusion

We have shown that the frequency of intra- and intersubtype HIV-1 recombination during reverse transcription is directly related to sequence identity of the parental HIV-1 isolates and is dependent on equal replicative fitness of both isolates in a dual infection. However, the preferential sites of recombination with specific HIV-1 isolates (e.g. at C3 region of 115-A virus) can alter this relationship between sequence identity and recombination frequency. As described, a specific RNA sequence/structure combination between two diverse HIV-1 isolates may result in a unique recombination "hotspot". Following multiple rounds of virus replication, less than 25% of these intra- or intersubtype recombinant genomes (in this case, *env *genes) are replication competent and survive the competition with parental HIV-1 isolates. These findings suggest that the initial selection of replication competency may preceded the selection based on transmission efficiency, host immune evasion, and many other parameters responsible for shaping the evolution of unique and circulating recombinant forms (URF and CRFs) of HIV-1 isolates found in the epidemic. The impact of selection based on replication efficiency versus the host-mediated selection is now being investigated using Ugandan samples.

## Methods

### Cells

293T cells and U87.CD4.CXCR4 cells were grown in Dulbecco's modified Eagle's medium supplemented with 10% fetal calf serum, penicillin, and streptomycin (from Invitrogen) and maintained at 37°C with 10% CO_2_. CD4 and CXCR4 expression in the U87 cell cultures was selected with 300 mg/mL Geneticin (G418) and 1 mg/mL puromycin (Life Technologies, Inc.), respectively. MT4 cells were maintained in RPMI 1640 medium supplemented with 10% fetal calf serum and antibiotics at 37°C with 5% CO_2_.

### Viruses

Five non-syncytium inducing (NSI) HIV-1 isolates were isolated from HIV-infected Ugandans, characterized for co-receptor usage, and subtyped based on phylogenetic sequence analyses as previously described [28]. Two primary subtype A HIV-1 isolates (115-A and 120-A) and three subtype D strains (89-D, 122-D, and 126-D) were selected. Due to previous confusion in strain nomenclature, we have modified the virus names from that previously published (A14 is now 115-A, A15 = 120-A, D13 = 122-D, D14 = 126-D, and D15 = 89-D) [28]. All HIV-1 isolates were syncytium-inducing (SI) and utilized the CXCR4-coreceptor (X4) for entry [28]. All viral stocks were previously propagated and expanded in PHA-stimulated, IL-2 treated peripheral blood mononuclear cells as described [28]. Tissue culture dose for 50% infectivity (TCID_50_) was determined for each isolate using the Reed and Muench method [45], and titers were expressed as infectious units per milliliter (IU/ml) [46]. All the *env *genes of these HIV-1 isolates have been previously sequenced [28], aligned, and have the following accession numbers: 115-A, 120-A, 89-D, 122-D, and 126-D.

### Single cycle tissue culture assay

Single cycle assays were completed using an assay previously developed by our laboratory [30,32]. HIV-1 envelope gene fragments from subtypes A and D (HXB2 nt 6420–7520) were PCR amplified from viral DNA and cloned in a pKS-derived Kn^r^/ORI plasmid following standard cloning techniques [32,47]. As previously described, we have designed two types of plasmids, pLac^+ ^and pLac^- ^plasmids carry, as genetic markers, either a functional *LacZ*' gene or a sequence complementary to the mRNA coding for *Escherichia coli malT *gene, respectively. These two genetic markers, lac^+ ^and lac^- ^in their respective plasmids are schematically represented in Figure 2B. All constructions were verified by sequencing. [48,49]Defective retrovirus particles were produced as described [30]. The medium was replaced 8 h after transfection, and the vector supernatants were recovered 36 h later. Non-internalized DNA was removed by treatment of the vector supernatants with DNaseI (1 μg/ml in the presence of 1 μM MgCl_2_) for 30 min at 37°C. Amount of p24 present in supernatants was determined by using the HIV-1 p24 enzyme-linked immunosorbent assay kit (PerkinElmer Life Sciences). When necessary, vector supernatants were concentrated by using Centricon^® ^YM-50 centrifugal filter devices (Amicon-Millipore) before transduction.

MT4 cells were transduced with 200 ng of p24 antigen per 10^6 ^cells (an approximate multiplicity of infection of 20) in 35-mm dishes in a 500-μl volume. Two hours post-transduction, the cells were diluted up to a 4-ml volume with supplemented RPMI medium and maintained at 37°C in a 5% CO_2 _incubator for 40 h. The reverse transcription products were purified by Hirt [50] as described [51]. The purified double stranded DNA was digested with DpnI for 2 h at 37°C (in order to eliminate possible contaminating DNA of bacterial origin) prior to PCR amplification (20 cycles) with primers BH and SH (Figure 2). The amplified product was purified after electrophoresis on agarose gel, digested with PstI and BamHI, ligated into an appropriate plasmid vector and transformed in *E.coli*. Plating on IPTG/X-Gal containing dishes allowed blue/white screening of recombinant and parental colonies, respectively [30].

### In Vitro recombination assays

In vitro recombination assays were performed using the reconstituted system previously developed in our laboratory [47]. RNA synthesis was performed as previously described [52]. RT purification and activity tests were carried out as described by Canard and colleagues [53]. Constructs used for RNA synthesis were generated following standard cloning procedures. Reverse transcription was carried out on the donor RNA (100 mM) in the presence of an equimolar amount of acceptor RNA after annealing an oligonucleotide specifically onto the donor template. Reverse transcription was started by the addition of HIV-1 RT at a final concentration of 400 nM and carried out for 60 min. Synthesis of the second DNA strand, BamHI and PstI digestion, ligation, and *E. coli *transformation were carried out as previously described [47]. The frequency of recombination is derived from the rate of blue colonies (recombinant) divided by the sum of blue plus white (parental) colonies.

### Measuring the frequency of recombination in the in vitro reverse transcription assay and single-cycle infection assay

To determine the frequency of recombination in the single cycle assay, the total number of blue and white colonies were calculated and applied in the equation as previously described [30]. The frequency of recombination (*F*) is calcuated by the equation F = b/(2/3 [N(n/48) + b]), where *N *and *b *are the total number of white and blue colonies, respectively. The recombination rates per nucleotide (*f*) within a given interval (*i*) is given by *f *= *F*(*x*_*i*_/*X*)/*z*, where *F *is as above, *x*_*i *_is the number of colonies analyzed where recombination was identified to have occurred within the interval considered, *X *is the total number of colonies on which mapping was performed, and *z *is the size in nucleotides of the interval. This takes into account the generation of heterozygous particles as described by the Hardy-Weinberg equation [38] and the bias introduced into the estimation of the frequency of recombination by cloning reverse transcription products from lac ^-/- ^(1/3) relative to lac ^+/- ^vectors [30]. For the calculation of the frequency of recombination in heterozygous particles, the total number of colonies must be multiplied by two-thirds. To accurately estimate the frequency of recombination, another factor to take into account is the background among the white colonies derived from cloning of cellular DNA which co-purified with the reverse transcription products. Typically, 48 white colonies are analyzed in each assay and a correction factor is established, given by *n*/48, where *n *is the number of colonies resulting from cloning of reverse transcription products (very rare).

### HIV-1 dual infection assay

Different pairs of two HIV-1 isolates were used to simultaneously infect PBMC as described previously [54]. We performed four separate dual infections of U87.CXC4.CD4 cells with two HIV-1 isolates at the same multiplicity of infection (i.e. 10^-2^:10^-2 ^or 10^-3^:10^-3^). Virus mixtures were added to adherent U87.CD4.CXCR4 cells (500,000/well) for 2 h at 37°C, 5% CO_2 _in DMEM complete medium. Supernatants and two aliquots of cells were harvested at peak virus production (typically day 10) as measured by reverse transcriptase activity in the supernatant [46,55]. Cells were resuspended in 10% DMSO/90% fetal bovine serum, and then stored at -80°C for subsequent analysis.

### PCR strategy to amplify HIV-1 recombinants in the multiple cycle assay

For all dual infection experiments, proviral DNA was extracted from lysed PBMC using the QIAamp DNA Blood Kit (Qiagen). A segment of the *env *genes of HIV-1 genome were PCR amplified using a set of universal primers: envB [56] – envN [57] for a ~3 Kbp fragment encoding the gp120 of *env*. Subtype-specific primers internal to the previous *env *products were then used to PCR amplify subtype A/D recombinants of using subtype- or isolate-specific primers, *a*115-envC1 (TAGTGCAGAAAAGCATAATG; a 115-A-specific sense primer), *d*-envC4 (TGTCAATTTCTCTTTCCCAC; a subtype-D specific antisense primer), *a*120-envC1 (AAGCATATGATGCAGAAGTAC; a 120-D specific sense primer), *d*-envC1 (TAAAACAGAGGCACATAATA; a subtype D specific sense primer), and *a*-envC4 (TGCTAATTTCTTTATCCCAT; a subtype A specific antisense primer). For intrasubtype dual infections, the following subtype-specific primers were used: *a*115-envC4 (CCTCTTGCCAAGAATGTTC; a 115-A antisense primer), *a*120-envC4 (TCTAGTGTCTGGACCGAT; a 120-D antisense primer), *d*122-envC1 (GTCAGGGCGAGCATACTA; 122-D sense primer), *d*122-envC4 (CCCAGTGGTTCAATCTC; a 122-D antisense primer), *d*126-envC1 (ACAAGGGCAAGCATGGTA; a 126-D sense primer), and *d*126-envC4 (GACCTAGTGGCTCAATTTTTAC; a 126-D antisense primer). All of these intrasubtype and intersubtype specific primer sets flanked the C1 to C4 regions of *env *(nt positions of approximately 6520 to 7740). Both external and nested PCR reactions were carried out in a 100-μl reaction mixture with defined cycling conditions [31]. PCR-amplified products were separated on agarose gels and then purified using the QIAquick PCR Purification Kit (Qiagen). Control PCR amplifications were performed with subtype-specific DNA templates to rule out the possibility of Taq-generated recombinants [58]. These isolate/subtype specific templates were generated by cloning the PCR amplified *env *gene of virus 115-A, 120-A, and 89-D, 122-D, and 126-D into pCR2.1 vector (Invitrogen). As an amplification/PCR quantitation control for recombination frequency calculations, plasmids containing entire *env *gene of the subtype A and D viruses were serially diluted and PCR amplified. Intensities of these products on agarose gels were then used as a standard curves and to calculate copy number of recombined viral RNA molecules in the dual infection based on the intensity of RT-PCR amplified products.

### Calculating the frequency of recombination in the multiple cycle dual infection assay

To determine the frequency of recombination after multiple rounds of infection and replication in tissue culture, external and nested PCR amplification were used with subtype or isolate specific primers as described above. The frequency of recombination was determined using both parental sequence primer sets (eg. *d*-envC1 – *d*-envC4), and recombinant primer sets (eg. *d*-envC1 – *a*115-envC1). The frequency of recombination was determined by quantifying the intensity of the PCR products on an agarose gel using Quantity One software (BioRad). Copy number of recombined and parental HIV-1 DNA is then derived by the intensity of the product compared to that of amplified product from known copy numbers of plasmid controls. The recombination frequency is calculated by dividing the total recombinant virus production by the total virus production in the system.

### Heteroduplex tracking assay for detection of two HIV-1 *env *fragments

Nested PCR products of the *env *gene were analyzed by heteroduplex tracking analysis [54]. The same genomic regions were PCR amplified from a subtype E HIV-1 *env *clone (E-pTH22) [59] for use as a DNA probe. For this amplification, the E80 primer was radiolabeled with T4 polynucleotide kinase and 2 μCi of [γ-^32^P]ATP and paired with the E125 primer to amplify the C2-C4 region of *env *[54]. The same pair of cold primers were employed to PCR amplify the HIV-1 *env *DNA from each dual infection. Radiolabeled PCR-amplified probes were separated on 1% agarose gels and purified with the Qiaquick gel extraction kit (Qiagen). Reaction mixtures contained DNA annealing buffer (100 mM NaCl, 10 mM Tris-HCl [pH 7.8], and 2 mM EDTA), 10 μl of unlabeled PCR-amplified DNA from the competition culture, and approximately 0.1 pmol of radioactive probe DNA [54]. The competition and probe DNA in this mixture was then denatured at 95°C for 3 min and then rapidly annealed on wet ice. After 30 min on ice, the DNA heteroduplexes were resolved on Tris-borate-EDTA buffer on 5 to 8% nondenaturing polyacrylamide gels (30:0.8 acrylamide-bisacrylamide) for 2.5 h at 200 V. The percentage of polyacrylamide in the gel matrix was dependent on the size of the amplified product employed in the heteroduplex tracking analysis. Gels were dried and exposed to X-ray film (Eastman Kodak Co., Rochester, N.Y.). Heteroduplexes representing production of each isolate in a dual infection were quantified with the Bio-Rad Phosphor-imager.

### Estimation of viral fitness

In our HIV-1 competition experiments, the final ratio of the two viruses produced from a dual infection was determined by heteroduplex tracking analysis and compared to production in the monoinfections. Production of individual HIV-1 isolates in a dual infection (*f*_0_) was divided by the initial proportion in the inoculum (*i*_*o*_) and is referred to as relative fitness (*w *= *f*_0_/*i*_0_) [54]. The ratio of the relative fitness values of each HIV-1 variant in the competition is a measure of the fitness difference (*W*_*D*_) between the two HIV-1 strains (*W*_*D *_= *w*_*M*_/*w*_*L*_), where *w*_*M *_and *w*_*L *_correspond to the relative fitness of the more and less fit virus, respectively [54]. As indicated in the text, viral fitness can also be calculated from the parental specific PCR products.

### Reverse transcription/strand transfer reactions

These reconstituted in vitro reverse transcription reactions focused on template switching in the C3 region of *env *using 115-A or 120-A donor RNA and 89-D acceptor RNA templates. For these reactions RNA transcripts were made from PCR products derived from the subtype clones. Primer pairs (5' **GATTTAGGTGACACTATAG***ATATA*ATGAGGTAGTCAAACAATTA-3' and 5'-TTTATTCTGCATTTGAGAGT-3' for 115-A, 5'-**GATTTAGGTGACACTATAG***ATATA*AGGAGGTAGCCAAACAATTA-3' and 5'-TTTATTCTGCATTGGAGAGT-3' for 120-A, and 5'-**GATTTAGGTGACACTATAG***TATAT*AGAATGGAATAAAACTATAC-3' and 5'-ACCGTTTGTGTTTGTACTCT-3' for 89-D) were used in PCR reactions to amplify nts 7255-7474 (relative to HXB-2 provirus numbering) for 115-A and 120-A, and 7235-7454 for 89-D. The bolded regions of the primers are SP6 promoter sequences while italicized regions are non HIV sequences. PCR products were recovered on native polyacrylamide gels and SP6 RNA polymerase was used to produce run-off transcripts of 225 nts. A DNA primer that binds specifically to the donor RNA transcript (5'-TTTATTCTGCATTTGAGAGT-3' or 5'-TTTATTCTGCATTGGAGAGT-3' for A-115 and A-102, respectively) was ^32^P-labeled at the 5' end with T4 polynucleotide kinase according to the manufacturer's protocol (New England Biolabs). The donor RNA was hybridized to a complementary labeled primer by mixing primer:transcript at approximately 3:1 ratio in 50 mM Tris-HCl (pH 8.0), 1 mM DTT, 80 mM KCl. The mixture was heated to 65°C for 5 min and then slowly cooled to room temperature. Donor RNA-primer DNA hybrids (2 nM final concentration of RNA) were preincubated for 3 min in the presence or absence of 10 nM acceptor RNA template and NC (as indicated) in 42 μl of buffer (see below) at 37°C. One molecule of NC per two nucleotides was used in the reactions. Wild-type and mutant NC proteins from the HIV-1 NL4-3 strain were prepared as explained previously[60,61]. The reactions were initiated by the addition of 8 μl of HIV-RT (80 nM final in reactions) to a mixture of 50 mM Tris-HCl (pH 8.0), 1 mM dithiothreitol, 80 mM KCl, 6 mM MgCl_2_, 100 μM dNTPs, 5 mM AMP (pH 7.0), 25 μM ZnCl_2 _and 0.4 units/μl RNase inhibitor. Reactions were allowed to incubate for 0, 30 s, 1, 2, 4, 8, 16, 32, and 64 min at 37°C prior to quenching a 6 μl aliquot of each reaction with 4 μl 25 mM EDTA (pH 8.0) and 2.5 ng of RNase-DNase free enzyme for 20 min at 37°C. Two μl of proteinase K at 2 mg/ml in 1.25% SDS, 15 mM EDTA (pH 8.0) and 10 mM Tris (pH 8.0) was then added to the above mixture, which was placed at 65°C for 1 hour. Finally, 12 μl of 2X formamide dye (90% formamide, 10 mM EDTA (pH 8.0), 0.1% xylene cyanol, 0.1% bromophenol blue) was added to the mixture and the samples were resolved on an 8% denaturing polyacrylamide gel containing 7 M urea. Extended DNA products were quantified by phosphorimager analysis using a Bio-Rad FX phosphoimager.

## Abbreviations

HIV-1 human immunodeficiency virus type-1

CRF circulating recombinant form

URF unique recombinant form

*env *HIV-1 envelope gene

dNTPs deoxynucleoside triphosphates

PCR polymerase chain reaction

MOI multiplicity of infection

TCID_50 _tissue culture dose for 50% infectivity

HTA heteroduplex tracking assay

RT reverse transcriptase

## Competing interests

The author(s) declare that they have no competing interests.

## Authors' contributions

**HB **was responsible for the majority of this manuscript and performed the research in the laboratories of Drs. Arts and Negroni as part of her Ph.D. dissertation. She was responsible for establishing the recombination systems in Figure 2A–C, and for the data presented in Figures 3, 4, and 5A. **YG and MA **performed the experimentation on the multiple cycle recombination system presented in Figure 2C and Figure 3B. **RG and VG **worked with Dr. Baird on the research performed at the Institut Pasteur and was responsible for some of the data in Figures 3, 4, and 5A. **ML **worked on a series of preliminary experiments leading to the data presented in Figure 5. **RA and DS **performed the experiments presented in Figure 5 and that were performed at the University of Maryland. **MN and EA **devised the concepts for these studies and supervised all of the research.
